# Simultaneous Determination of 54 Pesticides in Proso Millet Using QuEChERS with Liquid Chromatography-Tandem Mass Spectrometry (LC–MS/MS)

**DOI:** 10.3390/molecules28186575

**Published:** 2023-09-12

**Authors:** Chao Ding, Pengcheng Ren, Yanli Qi, Yanmei Yang, Shu Qin

**Affiliations:** Shanxi Center for Testing of Functional Agro-Products, Shanxi Agricultural University, Taiyuan 030031, Chinaymyangsuda@163.com (Y.Y.)

**Keywords:** proso millet, pesticides, determination, QuEChERS, LC–MS/MS

## Abstract

To assess the potential risks posed to the environment and human health, analyzing pesticide residues in proso millet is important. This paper aimed to develop a modified QuEChERS method with liquid chromatography-tandem mass spectrometry (LC–MS/MS) for the analysis of 54 pesticide residues in proso millet. Parameters including the mobile phase of the instrument, the acidity of the extraction solvent, and the type of absorbents were optimized to provide satisfactory performance. The method was validated concerning linearity, limit of quantification (LOQ), matrix effect, accuracy, and precision. In detail, the linearity of the matrix-matched calibration curve was acceptable with correlation coefficients (R^2^) higher than 0.99. The mean recovery was in the range of 86% to 114% with relative standard deviations (RSDs) ≤ 20% (n = 5). The LOQ was determined to be 0.25–10 μg/kg. The developed method was feasible for the determination of multiple pesticide residues in proso millet.

## 1. Introduction

Proso millet (*Panicum miliaceum* L.) is one of the featured coarse cereals in Shanxi, China. Due to its tolerance to drought and heat conditions as well as short growing period, proso millet is planted widely. In past years, proso millet has also received extensive attention because it is rich in protein, starch, fat, vitamins, and minerals [1,2]. Besides, natural active substances in proso millet, including polyphenols, phytic acid, and alkaloids, are beneficial for regulating blood sugar and lipids, as well as resisting oxidation [3]. Previous studies indicated that intake of proso millet and its processed product could help reduce the risk of chronic diseases, such as type 2 diabetes and liver damage [4,5,6,7].

In the cultivation of proso millet, pesticides are widely utilized to control disease, insect pests, and weeds, which is beneficial for improving its production and quality [8]. However, due to its low economic benefit, registered pesticide for proso millet is limited [9]. Widely used pesticides concerning organophosphorus, carbamate, pyrethroid, and nicotinoids may be applied during the cultivation of proso millet. Moreover, pesticides remaining in the soil can be uptaken and transported by crops, such as thiamethoxam, imidacloprid, and chlorpyrifos, azoxystrobin, acetamiprid [10,11]. The residue in crops will pose a toxicological impact on the environment and human health, especially for those with high toxicity, such as phorate [12]. Thus, developing a reliable method for determining multiple pesticide residues to monitor the dietary intake risk from proso millet is crucial. However, relevant studies are still limited.

As a cheap, easy, quick, safe method, QuEChERS has been the most commonly used sample preparation method since first developed in 2003, enabling the determination of multiple pesticides simultaneously [13]. It has been used for the analysis of multi-residues in various cereals, such as rice [14], corn [15], and wheat grains [16]. Ruan et al. also developed a method for the analysis of 34 pesticides in proso millet by QuEChERS [17]. However, in this study, QuEChERS was coupled with online gel permeation chromatography–gas chromatography, which was time-consuming and required labor. In comparison, liquid chromatography-tandem mass spectrometry (LC–MS/MS) is predominant due to its good sensitivity, selectivity, and short analysis time [18,19]. Based on the above discussion, the combination of QuEChERS with LC–MS/MS is an ideal strategy for detecting multiple pesticide residues in proso millet.

The purpose of this study was to develop and validate an efficient analytic method based on QuEChERS coupled with LC–MS/MS for the determination of multi-residue pesticides. In our study, 54 widely used pesticides, some of which were high-toxic or with a long residual period, were chosen as target compounds. The type of target pesticides includes acaricide, fungicide, herbicide, insecticide, and plant growth regulator. The developed method was proven to meet the requirements for the detection of multi-residues in proso millet.

## 2. Results and Discussion

### 2.1. Optimization of Instrumental Conditions

In this study, mass spectrum acquisition parameters of 54 pesticides were firstly optimized by direct injection of 0.1 mg/L individual standard solution in ACN to a mass spectrometer. The precursor and product ion were selected using the scan type of Q1 MS and product scan, respectively. Then collision energy (CE) and declustering potential (DP) were optimized based on the intensity of the ion. Most of the pesticides were detected using the positive mode while the other four pesticides including fipronil, fipronil desulfinyl, fipronil sulfone, and fipronil sulfide gave a better response using the negative mode. Detailed parameters were exhibited in Table 1. The product ion with a higher response was selected as a quantitative ion and the other one as a confirmative ion.

**Figure 1:**
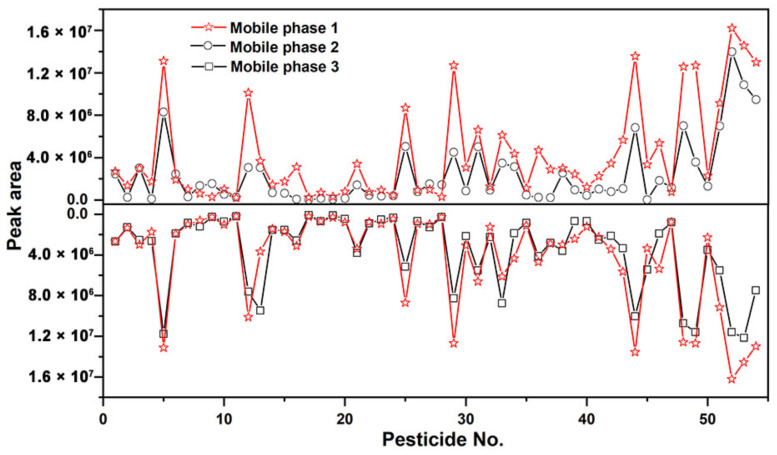
The peak response of 54 pesticides (0.1 mg/L) was obtained using different combinations of mobile phase.

**Figure 2:**
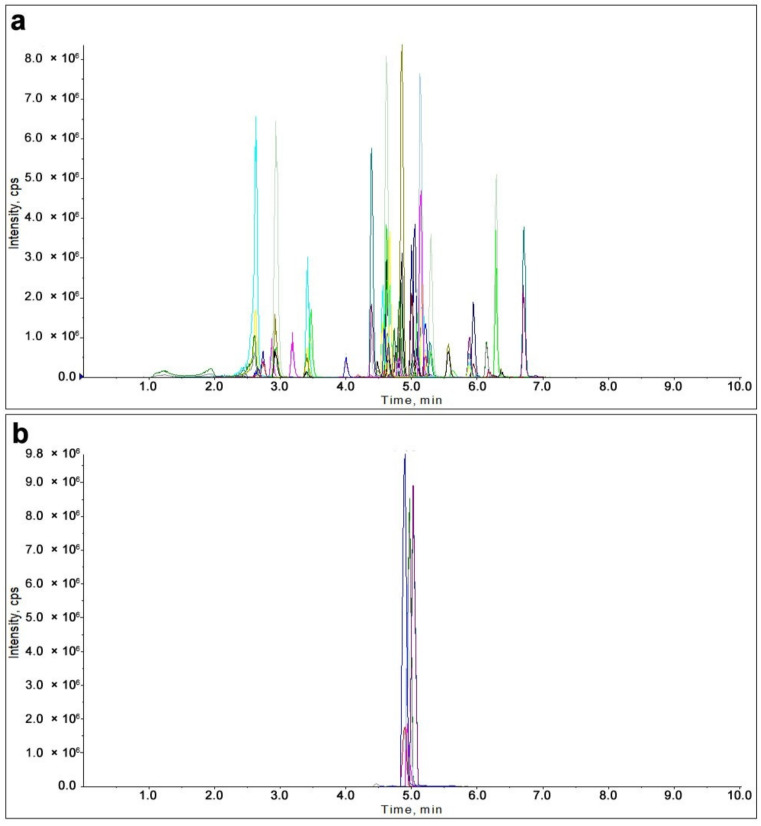
MRM chromatograms of 54 pesticides (0.1 mg/L) were obtained using the positive mode (**a**) and negative mode (**b**). The different colored lines correspond to different pesticides.

The mobile phase of LC Chromatographic played an important role in the ionization efficiency of pesticides [20]. The formic acid (FA) and ammonium acetate studied were recommended to be commonly used as MS-compatible additives [21]. In the following study, the effect of three different mobile combinations was investigated in terms of peak area: 0.1% formic acid (FA) aqueous solution containing 4 mM ammonium acetate + methanol (mobile phase 1); 0.1% formic acid (FA) aqueous solution containing 4 mM ammonium acetate + acetonitrile (mobile phase 2); 0.1% formic acid (FA) aqueous solution + methanol (mobile phase 3); According to results exhibited in Figure 1, compared with mobile phase 2 and 3, higher peak response of most pesticides was obtained when using mobile phase 1. Thus, mobile phase 1 was utilized for the separation of 54 target compounds in 10 min. It is noted that the peak shape or retention time of basic compounds is easily affected by the reversed-phase (RP) column due to overload behavior. However, McCalley indicated that reduced column efficiency occurs with sample amounts introduced onto RP columns of greater than 50 ng [22]. And the injection amount in our experiment was far less than the above value. The effect of different mobile on peak shape or retention time was not studied herein. The corresponding MRM chromatograms of 54 pesticides (0.1 mg/L) using the positive mode and negative mode were exhibited in Figure 2a,b.

### 2.2. Optimization of QuEChERS Method

#### 2.2.1. Extraction

Based on EN15662-2018, soaking the sample with water can improve the extraction efficiency of pesticides if the water content of the sample is <10% [23]. Therefore, 5 mL of water was added to homogenized proso millet samples and allowed to stand for 20 min before extraction.

**Figure 3:**
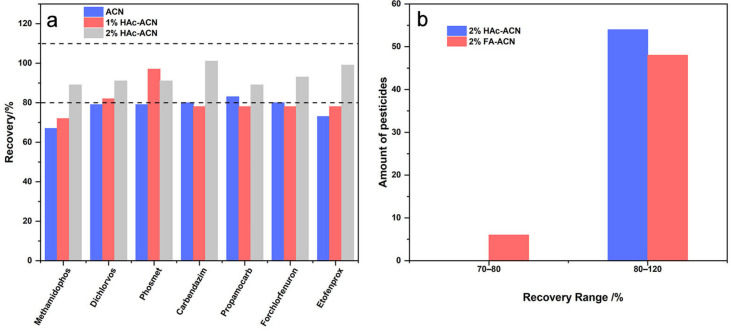
(**a**) Recoveries of methamidophos, dichlorvos, phosmet, carbendazim, propamocarb, forchlofenuron, and etofenprox extracted with pure ACN, 1% HAc-ACN and 2% HAc-ACN; The upper and lower dotted line indicated recoveries of 110% and 80%, respectively. (**b**) Amount of pesticides in different recovery range using 2% HAc-ACN and 2% FA-ACN as extract solvents.

The solvent is one of the main factors to affect the extraction efficiency. Due to its medium polarity, acetonitrile is the most common solvent used to determine various pesticides with different physicochemical properties [24,25]. The addition of acid could also influence the extraction ability of acetonitrile. In this section, the recovery of pure acetonitrile (ACN), acetonitrile with 1% acetic acid (1% HAc-ACN), and acetonitrile with 2% acetic acid (2% HAc-ACN) were compared. In detail, 50 μL of standard solution (10 mg/L) containing 54 pesticides was added into a blank proso millet sample. After standing for 30 min, samples were extracted with three different extract solvents. Recovery indicated that no obvious difference was shown for 48 pesticides. While for methamidophos, the recovery increased from 67% to 89% with the increase of HAc content in acetonitrile from 0% to 2%. Previous studies revealed that the increase in acid could improve the stability, which led to a significant increase in recovery [26]. An increase of acid in acetonitrile could also improve the extract efficiency of dichlorvos, phosmet, carbendazim, propamocarb, forchlorfenuron, and etofenprox. As shown in Figure 3a, the recovery of these five pesticides extracted with 2% HAc-ACN was all in the range of 80–110%, which was more satisfactory than pure ACN and 1% HAc-ACN. Thus, the concentration of acid in ACN was determined to be 2% and a further increase of acid will result in more interference, which can contaminate the instrument. In the following experiment, the type of acid was identified as the next variable to be optimized. The recovery of pesticides extracted with 2% HAc-ACN and acetonitrile with 2% formic acid (2% FA-ACN) was compared. Figure 3b shows that recoveries of 54 pesticides all ranged from 70–120% when extracted with 2% HAc-ACN and 2% FA-ACN. However, when using 2% FA-ACN as the extraction reagent, 6 of 54 pesticides had recoveries between 70–80%, whereas recoveries of 54 pesticides were all above 80% in the case of 2% HAc-ACN under the same conditions. Based on the above results, 2% HAc-ACN was selected as the best extract solvent.

#### 2.2.2. Clean-Up

The clean-up procedure is a critical step for the determination of pesticides. The optimum absorbent should enable satisfactory recovery as well as minimum interferences. Proso millet comprises complex components, including carbohydrates, proteins, fats, and dietary fibers. Thus, the optimization of absorbents to remove interferences is essential. Commonly used absorbents for purification include C_18_, PSA, and GCB. C_18_ is used to remove non-polar substances such as lipids and fats due to its large surface [27]. PSA, as a weak anion exchange filler, is used for the removal of fatty acids and sugars [28]. GCB with a large surface area exhibited a good clean-up effect on pigments [29]. In the following study, the recovery of 54 pesticides purified with different combinations of absorbents (1: 37.5 mg PSA + 225 mg MgSO_4_; 2: 37.5 mg PSA + 225 mg MgSO_4_ + 7.5 mg GCB; 3: 37.5 mg PSA + 225 mg MgSO_4_ + 50 mg C_18_; 4: 37.5 mg PSA + 50 mg C_18_ +7.5 mg GCB + 225 mg MgSO_4_) was investigated. The recovery of 54 pesticides was all within an acceptable range between 70–110% and no obvious difference was exhibited when using four kinds of absorbents [30,31]. Taking minimum interferences into consideration, a combination of 37.5 mg PSA + 50 mg C_18_ + 7.5 mg GCB + 225 mg MgSO_4_ was chosen as the best absorbent for the final method.

### 2.3. Method Validation

The optimized method was validated in terms of linear range, LOQ, matrix effect, accuracy, and precision. Standard solutions were prepared using matrix extract with a concentration from 0.004–0.2 mg/L. 54 pesticides exhibited a satisfactory linear relationship between peak area and concentration with a correlation coefficient (r^2^) > 0.99. LOQ was determined as the concentration with the signal-to-noise ratio (S/N) to be 10. As shown in Table 2, the calculated LOQ was in the range of 0.25 to 10 μg/kg.

Matrix effects (ME) were evaluated by comparing the slope of the matrix-matched and solvent-based calibration curves in triplicate. According to ME (%) value, there are two cases: (1) the matrix effect is ignored if the value is within the range from −20% to 20%; (2) the matrix effect is significant if the value is lower than -20% or higher than 20% [32]. ME (%) value of 54 pesticides are exhibited in Table 2. The results show that 19 pesticides exhibited ignored matrix effects, while 4 and 31 pesticides exhibited significant enhancement and suppression effects, respectively. The matrix effect of polar compounds (log Kow < 1) was reported to be more significant [33]. However, methamidophos, acephate, thiamethoxam, and methomyl exhibited an ignored matrix effect in our manuscript. It was suggested that the matrix effect was closely related to the sample type and extraction method [34]. To compensate for matrix effects, a matrix matrix-matched calibration curve was applied to obviate possible interferences for quantification in samples.

The accuracy and precision of the method were evaluated by spiking blank proso millet samples at 3 concentration levels with five replications: 0.01, 0.1, and 0.2 mg/kg. As shown in Table 2, recoveries of 54 pesticides were within the acceptable range from 86% to 114%. Precision was evaluated by relative standard deviation (RSDs, %), which was in the range of 1–20%. According to the Guideline for the Testing of Pesticide Residues in Crops (NY/T 788-2018) [30], the recovery criteria at spiking levels of 0.01, 0.1 and 0.2 mg/kg is 60%–120% (RSD ≤ 30%), 70%–120% (RSD ≤ 20%) and 70%–110% (RSD ≤ 15%), respectively. The recovery based on the EU’s criteria of method validation procedures (SANTE/11312/2021) is 70%–110% with RSD ≤ 20% [31]. The results of recovery and RSDs could meet the requirements of NY/T 788-2018 and SANTE/11312/2021.

All of the above data demonstrated that the optimized QuEChERS was reliable for the determination of 54 pesticides in the proso millet sample.

### 2.4. Real Sample Analysis

The optimized were utilized to screen and quantify pesticides in 50 samples, which were collected from local farmer’s fields. The results in Table 3 show that aldicarb sulfone and imidacloprid were detected in one batch of samples respectively, with concentrations of 0.022 and 0.011 mg/kg, while the other 52 pesticides were not detected in all samples. The residue of aldicarb sulfone might have resulted from the application of aldicarb during planting. Maximum residue levels (MRLs) of pesticides in proso millet are not prescribed in China [35]. However, MRLs of aldicarb and imidacloprid in other similar grains, such as millet, are 0.02–0.05 mg/kg therefore, it might pose a residue risk on the consumers of proso millet. Furthermore, no pesticide product for proso millet is registered according to China Pesticide Information Network. Thus, relevant work is suggested to monitor the dietary intake risk of pesticides on proso millet.

## 3. Materials and Methods

### 3.1. Chemicals and Reagents

The certified reference materials of 54 pesticides (Table 1) in this work (≥98.0%) were purchased from the Agro-environmental Quality Supervision, Inspection & Testing Center (Tianjin, China). Methanol (HPLC grade) was obtained from Merck (Darmstadt, Germany). Acetonitrile (HPLC grade) was purchased from Tedia Company (Fairfield, OH, USA). Acetic acid (HPLC grade) and formic acid (HPLC grade) were from Fisher Regent Company (Beijing, China). Ammonium acetate (HPLC grade) was from Tianjin Guangfu Fine Chemical Research Institute (Tianjin, China), QuEChERS extraction salts pachets (4 g magnesium sulfate, 1 g sodium chloride, 1 g sodium citrate and 0.5 g disodium citrate sesquihydrate) were provided by Shimadzu Corporation. Anhydrous magnesium sulfate (MgSO_4_) (>99.0%) was from Sinopharm Chemical Reagent Co., Ltd. (Beijing, China). Primary secondary amine (PSA, 40–60 μm), graphitized carbon black (GCB, 120–400 Mesh), and octadecylsilane (C_18_, 50 μm, 60 A) were from Agela Technologies Inc. (Tianjin, China).

### 3.2. Sample Pretreatment

A 5.0 g homogenized sample was weighed into a 50 mL centrifuge tube. Then 5 mL of water was added to the sample and allowed to stand for 20 min. Afterwards, 10 mL of 2% of HAc in ACN was added and the mixture was then vortexed at 2500 rpm for 10 min. Subsequently, QuEChERS extraction salts were added, and the tubes were vortexed for another 5 min. The sample was then centrifuged at 8000 rpm for 3 min and the upper layer was collected.

1.5 mL of the upper organic layer was transferred into a 2 mL centrifuge tube containing 225 mg MgSO_4_, 50 mg C_18_, 7.5 mg GCB, and 37.5 mg PSA. Then the mixture was vortexed at 2500 rpm for 5 min followed by centrifugation at 5000 rpm for 2 min. Finally, 1.5 mL of the upper layer was filtered through a 0.22 µm nylon syringe filter and transferred into autosampler vials.

### 3.3. LC–MS/MS Analysis

Analysis of 54 pesticides was performed on Triple Quad 4500 (AB SCIEX, Framingham, MA, USA). Separation of target compounds was achieved on Waters ACQUITY UPLC^®^BEH C18 column (100 mm × 2.1 mm, 1.7 μm) at a temperature of 40 °C. The mobile phase consisted of 0.1% formic acid (FA) aqueous solution containing 4 mM ammonium acetate (A) and methanol (B). Gradient elution procedure was: 5–20% B at 0–1 min, 20–40% B for 0.10 min, 40–60% B for 1.9 min, 60–80% B for 0.1 min, 80–95% B for 1.9 min and holding for 2 min, 95–5% B for 2 min then holding for 1 min. The flow rate of the mobile phase was 0.30 mL/min and the injection volume was 2 μL.

Mass spectrometry analyses of pesticides were conducted in both positive mode (ESI^+^) and negative mode (ESI^−^). Mass spectrometry parameters were set as follows: curtain gas 35 psi, collision gas 9, ion source gas 1 (GS1) 55 psi, ion source gas 2 (GS2) 55 psi, ionspray voltage 5500 V (ESI^+^) and 4500 V (ESI^−^), temperature 550 °C. MRM parameters and retention time of 54 pesticides are shown in Table 1.

### 3.4. Method Validation

Method validation concerning linearity, matrix effect (ME), limit of quantification (LOQ), accuracy, and precision, was conducted according to SANTE/11312/2021 guidelines [31]. In detail, linearity was performed by preparing matrix-matched standards at concentrations of 0.004, 0.01, 0.02, 0.04, 0.1, and 0.2 mg/L. The ME was evaluated by comparing the slope of matrix-matched calibration curve and solvent-based calibration curve according to the following equation:

$$ME\%=\left(\frac{Slope\;of\;matrix\;matched\;calibration\;curve}{Slope\;of\;solvent\;based\;calibration\;curve}-1\right)\times \%$$

The accuracy and precision were evaluated by spiking the proso millet sample at three levels of 0.01, 0.1, and 0.2 mg/kg with five replications. The limit of quantification (LOQ) of 54 pesticides was determined as the concentration with the signal-to-noise ratio (S/N) to be 10.

## 4. Conclusions

In this paper, QuEChERS-UPLC-MS/MS method was developed and validated for the simultaneous detection of multi-class pesticides in proso millet. A series of optimizations were carried out in terms of chromatographic conditions, extraction, and purification. Satisfactory validation results including linearity, matrix effect, LOQs, accuracy, and precision, were obtained for all target pesticides. The method was applied for the analysis of 50 samples, which demonstrated its feasibility for monitoring pesticide residue in proso millet.

## Figures and Tables

**Table 1:** Category and mass spectrum acquisition parameters of 54 pesticides.

No.	Pesticide	Category ^1^	RT	Parent Ion	Product Ion ^2^	DP	CE
	Positive Mode						
1	Methamidophos	I	2.34	142.0	94.0 *; 125.0	57	19; 18
2	Phorate	I	5.54	261.0	75.0 *; 47.0	51	21; 53
3	Omethoate	I	2.76	214.0	183.0 *; 109.0	60	16; 36
4	Dichlorvos	I	4.62	221.0	109.0 *; 127.0	70	23; 27
5	Triazophos	I	5.07	314.1	162.1 *; 119.1	80	24; 50
6	Dimethoate	I	3.71	230.0	199.0 *; 125.0	56	13; 29
7	Chlorpyrifos	I	6.11	349.9	197.9 *; 97.0	75	28; 45
8	Acephate	I	2.68	184.0	143.0 *; 125.0	50	12; 25
9	Malathion	I	5.03	331.0	127.0 *; 99.0	70	16; 32
10	Phosalone	I	5.48	368.0	182.0 *; 322.0	76	20; 13
11	Phosmet	I	4.90	318.0	160.0 *; 133.0	61	17; 49
12	Isocarbophos	I	5.20	231.0	121.0 *; 109.0	100	26; 38
13	Diazinon	I	5.42	305.1	169.0 *; 153.1	90	28; 28
14	Profenofos	I	5.81	372.9	302.9 *; 344.9	80	25; 18
15	Phorate Sulfone	I	4.78	293.0	97.0 *; 115.0	65	50; 35
16	Phorate Sulfoxide	I	4.74	277.0	199.0 *; 153.0	25	13; 19
17	Tau-Fluvalinate	I	6.53	503.1	208.1 *; 181.1	61	16; 38
18	Iprodione	F	5.26	330.0	245.0 *; 288.0	30	20; 18
19	Deltamethrin	I	6.37	523.0	280.9 *; 506.0	55	23; 16
20	Fenpropathrin	I	6.11	350.2	125.1 *; 97.1	85	23; 46
21	Triadimefon	F	5.07	294.1	197.1 *; 225.1	70	21; 17
22	Aldicarb	I	4.30	116.1	89.0 *; 70.0	25	14; 14
23	Aldicarb Sulfone	I	2.90	240.1	148.0 *; 166.1	30	17; 16
24	Aldicarb Sulfoxide	I	2.82	207.1	132.0 *; 89.0	55	9; 20
25	Carbofuran	I	4.63	222.1	165.1 *; 123.0	70	16; 29
26	3-Hydroxy Carbofuran	I	3.63	238.1	181.1 *; 163.1	70	16; 18
27	Methomyl	I	3.05	163.1	88.0 *; 106.0	38	12; 14
28	Carbaryl	I	4.69	202.1	145.1 *; 127.1	56	15; 40
29	Carbendazim	F	3.06	192.1	160.1 *; 132.1	80	25; 41
30	Phoxim	I	5.41	299.1	129.0 *; 153.0	55	18; 10
31	Pyridaben	A	6.47	365.1	309.1 *; 147.1	77	17; 34
32	Pyrimethanil	F	4.97	200.1	183.1 *; 168.1	30	33; 40
33	Difenoconazole	F	5.55	406.1	251.0 *; 337.0	105	35; 24
34	Acetamiprid	I	3.64	223.1	126.0 *; 99.0	65	28; 60
35	Imidacloprid	I	3.38	256.1	175.1 *; 209.1	45	27; 22
36	Dimethomorph	F	5.01	388.1	301.1 *; 165.1	105	29; 43
37	Pendimethalin	H	6.16	282.1	212.1 *; 194.1	40	15; 28
38	Azoxystrobin	F	4.86	404.1	372.1 *; 344.1	80	20; 34
39	Thiamethoxam	I	3.10	292.0	211.1 *; 181.1	30	16; 30
40	Chlorfluazuron	I	6.32	540.0	382.9 *; 384.9	70	30; 30
41	Prochloraz	F	5.45	376.2	308.0 *; 266.0	20	15; 22
42	Chlorbenzuron	I	5.33	309.0	156.0 *; 139.0	50	18; 40
43	Diflubenzuron	I	5.24	311.0	158.0 *; 141.0	45	20; 49
44	Propamocarb	F	2.77	189.2	102.1 *; 74.0	70	24; 34
45	Forchlorfenuron	R	4.83	248.1	129.0 *; 93.0	50	23; 47
46	Etofenprox	I	6.90	394.2	177.1 *; 107.0	30	19; 59
47	Chlorantraniliprole	I	4.84	484.0	285.9 *; 452.9	45	19; 25
48	Pyraclostrobin	F	5.38	388.1	194.1 *; 163.1	50	18; 36
49	Metalaxyl	F	4.82	280.2	220.1 *; 192.1	75	18; 24
50	Paclobutrazol	R	5.02	294.1	70.0 *; 125.0	90	40; 45
	Negative Mode						
51	Fipronil	I	5.17	434.9	330.0 *; 250.0	−25	−24; −38
52	Fipronil Desulfinyl	I	5.12	387.0	351.0 *; 282.0	−30	−19; −47
53	Fipronil Sulfone	I	5.26	450.9	414.9 *; 282.0	−28	−26; −38
54	Fipronil Sulfide	I	5.20	418.9	262.0 *; 383.0	−20	−35; −22

^1^ A acaricide, F fungicide, H herbicide, I insecticide, R plant growth regulator. ^2,^ * refers to Quantitative ion.

**Table 2:** Method limit of quantification, precision of 54 pesticides in proso millet.

Pesticide	R^2^	LOQ μg/kg	ME (%)	Recovery, % (RSD, %)		
				0.01 mg/kg	0.1 mg/kg	0.2 mg/kg
Methamidophos	0.9996	3.3	−5.7	86 (2)	89 (2)	89 (2)
Phorate	0.9900	2.0	6.8	95 (17)	85 (19)	105 (5)
Omethoate	0.9987	0.50	−18.0	98 (3)	100 (2)	94 (6)
Dichlorvos	0.9932	3.3	−18.5	93 (3)	91 (6)	95 (4)
Triazophos	0.9900	2.0	−36.4	103 (3)	102 (6)	107 (3)
Dimethoate	0.9975	1.7	−9.8	101 (3)	101 (2)	98 (2)
Chlorpyrifos	0.9970	4.0	−29.0	94 (5)	98 (5)	100 (5)
Acephate	0.9986	6.7	−8.0	99 (5)	92 (7)	98 (3)
Malathion	0.9905	1.0	−45.7	99 (7)	98 (17)	100 (5)
Phosalone	0.9977	2.5	−38.4	99 (11)	94 (10)	104 (6)
Phosmet	0.9939	10.0	−43.4	92 (5)	91 (11)	96 (6)
Isocarbophos	0.9943	5.0	−48.0	103 (4)	101 (3)	101 (1)
Diazinon	0.9976	3.3	−17.0	101 (4)	106 (6)	100 (2)
Profenofos	0.9994	3.3	−32.0	103 (8)	100 (7)	101 (7)
Phorate Sulfone	0.9904	0.67	−32.3	105 (3)	100 (6)	101 (7)
Phorate Sulfoxide	0.9929	0.50	−21.7	106 (8)	102 (5)	108 (1)
Tau-Fluvalinate	0.9909	0.33	−32.1	100 (11)	93 (9)	99 (11)
Iprodione	0.9989	2.0	−41.5	102 (8)	106 (2)	97 (2)
Deltamethrin	0.9960	8.3	−28.6	104 (8)	104 (5)	92 (14)
Fenpropathrin	0.9956	2.0	−29.7	97 (12)	104 (6)	104 (4)
Triadimefon	0.9994	5.0	−10.7	107 (4)	96 (4)	103 (2)
Aldicarb	0.9981	0.50	1.7	103 (11)	100 (4)	105 (2)
Aldicarb Sulfone	0.9994	3.0	−10.2	107 (7)	102 (4)	100 (6)
Aldicarb Sulfoxide	0.9972	0.033	−11.9	104 (10)	100 (9)	97 (8)
Carbofuran	0.9968	1.0	−32.4	105 (8)	109 (2)	104 (3)
3-HydroxyCarbofuran	0.9991	2.0	−26.1	103 (3)	104 (3)	100 (5)
Methomyl	0.9999	0.67	3.3	98 (5)	105 (3)	105 (1)
Carbaryl	0.9946	5.0	−30.2	95 (11)	96 (13)	105 (4)
Carbendazim	0.9995	0.67	−26.5	99 (2)	101 (2)	96 (2)
Phoxim	0.9982	10.0	−24.3	99 (3)	93 (1)	102 (3)
Pyridaben	0.9981	1.0	−43.3	102 (8)	114 (4)	107 (1)
Pyrimethanil	0.9986	2.5	−34.9	104 (8)	94 (5)	100 (4)
Difenoconazole	0.9970	2.0	−31.7	106 (5)	109 (4)	107 (3)
Acetamiprid	0.9987	0.50	−23.4	100 (2)	101 (2)	107 (3)
Imidacloprid	0.9978	2.0	−44.3	104 (3)	101 (3)	96 (3)
Dimethomorph	0.9970	3.3	−4.4	100 (6)	96 (5)	96 (4)
Pendimethalin	0.9955	10.0	30.6	95 (6)	100 (6)	94 (11)
Azoxystrobin	0.9983	2.5	−0.8	93 (10)	99 (7)	104 (4)
Thiamethoxam	0.9998	1.0	−14.1	100 (5)	105 (5)	100 (5)
Chlorfluazuron	0.9998	0.25	−39.4	106 (5)	99 (5)	95 (7)
Prochloraz	0.9976	0.50	21.0	90 (20)	99 (4)	96 (9)
Chlorbenzuron	0.9972	1.0	−40.9	102 (5)	103 (3)	103 (5)
Diflubenzuron	0.9993	2.0	−39.6	102 (7)	101 (3)	106 (2)
Propamocarb	0.9974	0.40	10.1	83 (2)	89 (3)	90 (5)
Forchlorfenuron	0.9982	2.0	−67.3	98 (3)	93 (4)	96 (2)
Etofenprox	0.9947	1.0	6.9	102 (7)	99 (5)	100 (5)
Chlorantraniliprole	0.9980	2.0	−57.2	98 (8)	104 (5)	85 (4)
Pyraclostrobin	0.9924	1.0	−29.0	103 (8)	98 (5)	101 (3)
Metalaxyl	0.9964	1.0	−40.0	105 (4)	102 (4)	100 (4)
Paclobutrazol	0.9963	3.3	−54.0	104 (7)	101 (4)	102 (3)
Fipronil	0.9946	1.0	58.6	99 (1)	103 (4)	103 (1)
Fipronil Desulfinyl	0.9935	2.0	−2.7	105 (1)	102 (2)	106 (1)
Fipronil Sulfone	0.9958	2.0	−4.9	102 (1)	105 (2)	105 (1)
Fipronil Sulfide	0.9936	1.0	23.0	106 (1)	103 (1)	106 (1)

**Table 3:** Detection of pesticide residue in proso millet samples (n = 50) collected from a local field.

Sample No.	Pesticide	Concentration (mg/kg)	MRL (mg/kg)
7	Aldicarb sulfone	0.022	NA ^1^
25	Imidacloprid	0.011	NA ^1^

^1^ Not Available.

## Data Availability

Not applicable.
